# Cultural food practices and sources of nutrition information among pregnant and postpartum migrant women from low- and middle-income countries residing in high income countries: A systematic review

**DOI:** 10.1371/journal.pone.0303185

**Published:** 2024-05-09

**Authors:** Bolanle R. Olajide, Paige van der Pligt, Fiona H. McKay

**Affiliations:** 1 Institute for Health Transformation (IHT), School of Health and Social Development, Deakin University, Burwood, Victoria, Australia; 2 Institute for Physical Activity and Nutrition (IPAN), School of Exercise and Nutrition Sciences, Deakin University, Geelong, Victoria, Australia; 3 Department of Nutrition, Western Health, Footscray, Australia; University of Greenwich, UNITED KINGDOM

## Abstract

Women in low- and middle-income countries (LMICs) may engage in a range of cultural food practices during pregnancy, including restricting or avoiding foods high in protein and iron, and foods rich in vitamins and minerals. While research has explored the cultural food practices of pregnant women in LMICs, there is less understanding of the continued cultural food practices of women who migrate to high-income countries and then become pregnant. This systematic review explores the existing research on cultural food practices and sources of nutrition information among pregnant and postpartum migrant women from LMICs, residing in high-income countries. A systematic search was conducted in April 2024 across Global Health, CINAHL, and MEDLINE, published in English, with no date restrictions. Eligible studies included those focused on pregnant and postpartum women who had migrated from LMICs to high-income countries. Studies were excluded if they comprised of non-immigrant women or did not involve LMIC participants. Screened were studies for eligibility, data were extracted, and study quality was assessed. In total, 17 studies comprising qualitative (n = 10) and quantitative (n = 7) approaches were included. In 14 studies participants adhered to cultural food practices, wherein certain nutritious foods were restricted during pregnancy or the postpartum period; three studies noted limited adherence due to support, acculturation, and access to traditional foods. Most studies (n = 10) reported traditional “hot” and “cold” food beliefs during pregnancy and postpartum, aiming to maintain humoral balance for maternal and child health and to prevent miscarriage. Nutrition advice was sought from family members, friends, relatives, healthcare providers, and media sources, with a preference for advice from family members in their home countries. There is a need for culturally appropriate nutrition education resources to guide pregnant migrants through healthy and harmful cultural food practices and overall nutrition during this crucial period. (PROSPERO Registration: CRD42023409990).

## Introduction

Women play a vital role in preserving cultural heritage by upholding traditional food practices, especially during pregnancy, where cultural food traditions influence food choices [1]. Studies conducted in low- and middle-income countries (LMICs) [2–4] have revealed the scope of cultural food practices women engage in specific to their cultures or groups. These food practices include avoiding foods such as eggs (believed to cause jaundice or hair loss in babies), milk (believed to cause vomiting and heartburn in babies after birth), restricting protein-rich foods (believed to generate heat and potentially cause miscarriage), and reducing the quantity of food consumed (to prevent complications that might arise from delivering a larger baby). These practices highlight the role of cultural traditions in shaping the dietary choices of pregnant women.

Culture plays an important role, not only in influencing the dietary choices of pregnant women, but also in shaping their transition to motherhood. The wide reaching impact of cultural beliefs and practices surrounding food choices during pregnancy highlight the significance of this transition. A review of maternal nutrition during pregnancy in LMICs revealed that cultural beliefs can act as a barrier to adequate nutrition during pregnancy [5]. Findings from studies conducted in a range of LMICs, including Pakistan [2], South Africa [4], and India [6], show that the cultural food practices that pregnant women engage in can lead to restrictions of essential nutrients crucial for the health of both the mother and fetus. A recent review [7] of women in Kenya and Indonesia found they restricted food including meat, eggs, and fruit, during pregnancy as a way to avoid difficult deliveries and reduce the risk of caesarean sections [7]. However, it is worth noting that in some cultural contexts, there exists a belief that the avoidance or consumption of specific foods contributes to the health of both mothers and fetus [8, 9]. A review by de Diego-Cordero and colleagues [7] emphasized that women who adhere to such cultural food practices firmly believe that disregarding them could be detrimental to the health of the fetus or the mother’s overall health. However, it is vital to understand practices from different regions and countries to continue supporting healthy practices, and knowing harmful practices and understanding trusted resources are essential to change harmful practices.

Apart from the influence of culture on the food choices of pregnant women, their sources of nutrition information can have a large influence on food choices during pregnancy. Like women from high income countries, women from LMICs often rely on advice from various sources such as family, friends, antenatal care, and their community for nutritional guidance [10, 11]. However, family members, particularly grandmothers and mothers, play a significant role in the dietary choices of pregnant women in LMIC. A review of the traditional pregnancy diets of migrant women from LMICs in their destination countries show that even after migration, women continue to rely on advice from family and female relatives, both in the destination country and home country [1].

Inadequate nutrient intake during pregnancy is a contributing factor to complications in pregnancy [10, 12]. Inadequate intake of nutrients during pregnancy can result from restrictions or the avoidance of certain foods [12]. A review exploring associations between maternal nutritional status and birth outcomes among African women revealed that insufficient nutrient intake by mothers is associated with poor maternal outcomes, including risk of gestational diabetes mellitus, pre-eclampsia, antepartum hemorrhage, postpartum hemorrhage, prolonged labor, and birth trauma, which can have both short- and long-term consequences [13]. Inadequate nutrient intake during pregnancy negatively affects fetal health and can lead to neural tube and congenital heart defects as well as poor fetal growth [14, 15]. In addition, studies indicate that poor maternal nutrient intake during pregnancy significantly raises the risk of offspring developing chronic health conditions later in life, such as diabetes, obesity, heart disease, and non-communicable diseases [16, 17].

While numerous studies have explored the food practices of pregnant women in LMICs [2–4, 11, 18, 19], the cultural food practices and sources of nutrition information of migrants from LMICs residing in high-income countries, both during pregnancy and the postpartum period, are less clear. This systematic review aims to synthesize the evidence exploring the cultural food practices and sources of nutrition information among pregnant and postpartum migrant women from LMICs residing in high-income countries. This review will also examine the sociodemographic factors that influence cultural food practices during pregnancy and postpartum. This review will significantly contribute to the literature by shedding light on an overlooked aspect of maternal nutrition, while also supporting the future development of culturally appropriate interventions aimed at improving maternal and fetal health outcomes among migrant populations.

## Method

A systematic search of three electronic databases, Global Health, CINAHL, and MEDLINE, was conducted in April 2024. The literature search was limited to three databases for several reasons. Firstly, these three specific databases are widely recognized as comprehensive sources within the field. Secondly, these databases were selected based on researchers’ preliminary research which indicated that these databases contain extensive coverage of relevant academic journals and scholarly publications pertinent to the research topic. Additionally, limiting the search to three databases allowed researchers to efficiently manage the scope of this review while still ensuring access to a diverse range of scholarly sources. While researchers recognize that other databases exist, we are confident that the chosen databases provided a robust foundation for this review. The key search terms included cultural, food practices, pregnancy, immigrants, and countries (low- and middle-income and high income). The Boolean operators “AND” and “OR” were used in conjunction with truncation operators and phrase searching. The search syntax was customized for each database. Refer to Table 1 for a comprehensive overview of the complete search strategy. This review has been registered with the international prospective register of systematic reviews (PROSPERO: CRD42023409990).

**Table 1:** Complete search strategy.

Concept	Search terms
1. Cultural	"cultural" OR (MH "Taboo") OR “Traditional belief*” OR “cultural belief*” OR “cultural practice*” OR “cultural food*” OR cultur*
2. Food Practices	“food practice*” OR “food belief*” OR “food taboo*” OR “food restriction*” OR “food tradition*” OR “food avoidance” OR “dietary belief*” OR “dietary practice*” OR “dietary restriction*” OR “perinatal belief*” OR “acculturation” OR “food prohibit*” OR “maternal nutrition” OR “nutrition intake*” OR “meal habit*” OR “Food habit*” OR “dietary intake*” OR “dietary pattern*” OR “food intake*”
3. Pregnancy	(mh pregnancy+) OR (mh expectant mothers) OR “pregnan*” OR “gestation*” OR “maternal*” OR “postnatal” OR “post natal” OR “postpartum” OR “post partum” OR “Recently Deliver*” OR “parent*” OR “pregnant women” OR “pregnant mother” OR “pregnancy diet” OR “maternal health” OR “pregnant immigra*”
4. Immigrant	(MH "Emigrants and Immigrants") OR (MH "Women") OR “Emigrant* and Immigrant*” OR “migrant*” OR foreigner* OR “emigrant*” OR “immigrant health” OR “migrant women” OR refugee* OR “immigra*” OR “immigrant pregnant*” OR “immigrant women”
5. Low- and Middle-Income countries	“low- and middle-incomecountr*” or “low middle income countr*” or LMIC or Afghan* or Albania* or Algeria* or Angola* or Argentina* or Armenia* or Azerbaijan* or Bangladesh* or Belarus* or Beliz* or Benin* or Bhutan* or Bolivia* or Bosnia* or Herzegovin* or Botswan* or Brazil* or Bulgaria* or Burkina* or Burundi* or “Cabo Verde*” or “Cape Verde*” or Cambodia* or Cameroon* or “Central African” or Chad* or China or Chinese or Colombia* or Comor* or Congo* or “Costa Rica*” or “Ivory Coast” or Cuba* or Djibouti* or Dominica* or Ecuador* or Egypt* or “El Salvador*” or Eritrea* or Eswatini* or Ethiopia* or Fiji* or Gabon* or Gambia* or Georgia* or Ghana* or Grenad* or Guatemala* or Guinea* or Guyan* or Haiti* or Hondura* or Hungar* or India* or Indonesia* or Iran* or Iraq* or Jamaica* or Jordan* or Kazakhstan* or Kenya* or Khmer or Kiribati* or Korea* or Kosov* or Kyrgyz* or Lao* or Leban* or Lesotho* or Liberia* or Libya* or Macedonia* or Madagascar* or Malawi* or Malaysia* or Maldiv* or Mali* or “Marshall Island*” or Mauritania* or Mauriti* or Mexic* or Micronesia* or Moldova* or Mongolia* or Montenegr* or Morocc* or Mozambi* or Myanma* or Burmese or Namibia* or Nauru* or Nepal* or Nicaragua* or Niger* or Nigeria* or Pakistan* or Palau* or Panama* or “Papua New Guinea*” or Paraguay* or Peru* or Philippines or Filipino or Romania* or Russia* or Rwanda* or Samoa* or “Sao Tome*” or Senegal* or Serbia* or Seychell* or “Sierra Leon*” or “Solomon Island*” or Somalia* or “South Africa*” or Sudan* or “Sri Lanka*” or “St Lucia*” or “Saint Lucia*” or “Saint Vincent” or “St Vincent” or Grenadines or Surinam* or Swazi* or Syria* or Tajikistan* or Tanzania* or Thai* or Timor* or Togo* or Tonga* or Tunisia* or Turk* or Turkmenistan* or Tuvalu* or Uganda* or Ukrain* or Uzbekistan* or Vanuatu* or Venezuela* or Vietnam* or “West Bank” or Gaza or Yemen* or Zambia* or Zimbabwe*
6. High income countries	“High income countr*” OR “Developed countr*” OR Andorra* OR “Antigua and Barbuda*” OR Aruba* OR Australia* OR Austria* OR Bahamas* OR Bahrain* OR Barbados* OR Belgium* OR Bermuda* OR “British Virgin Islands*” OR “Brunei Darussalam*” OR Canada* OR “Cayman Islands*” OR “Channel Islands*” OR Chile* OR Croatia* OR Curaçao* OR Cyprus* OR “Czech Republic*” OR Denmark* OR Estonia* OR “Faroe Islands*” OR Finland* OR France* OR “French Polynesia*” OR Germany* OR Gibraltar* OR Greece* OR Greenland* OR Guam* OR “Hong Kong*” OR Hungary* OR Iceland* OR Ireland* OR “Isle of Man*” OR Israel* OR Italy* OR Japan* OR Korea* OR Kuwait* OR Latvia* OR Liechtenstein* OR Lithuania* OR Luxembourg* OR Macao* OR Malta* OR Monaco* OR Nauru* OR Netherlands* OR “New Caledonia*” OR “New Zealand*” OR “Northern Mariana Islands*” OR Norway* OR Oman* OR Panama* OR Poland* OR Portugal* OR “Puerto Rico*” OR Qatar* OR Romania* OR “San Marino*” OR “Saudi Arabia*” OR Seychelles* OR Singapore* OR “Sint Maarten*” OR “Slovak Republic*” OR Slovenia* OR Spain* OR “St. Kitts and Nevis*” OR “St. Martin*” OR Sweden* OR Switzerland* OR “Taiwan China*” OR “Trinidad And Tobago*” OR “Turks and Caicos Islands*” OR “United Arab Emirates*” OR “United Kingdom*” OR “United States*” OR Uruguay* OR “Virgin Islands*”
	Concept 1, 2, 3, 4, 5,6 combined with AND
Limits	No Limits

### Study selection

**Fig 1:**
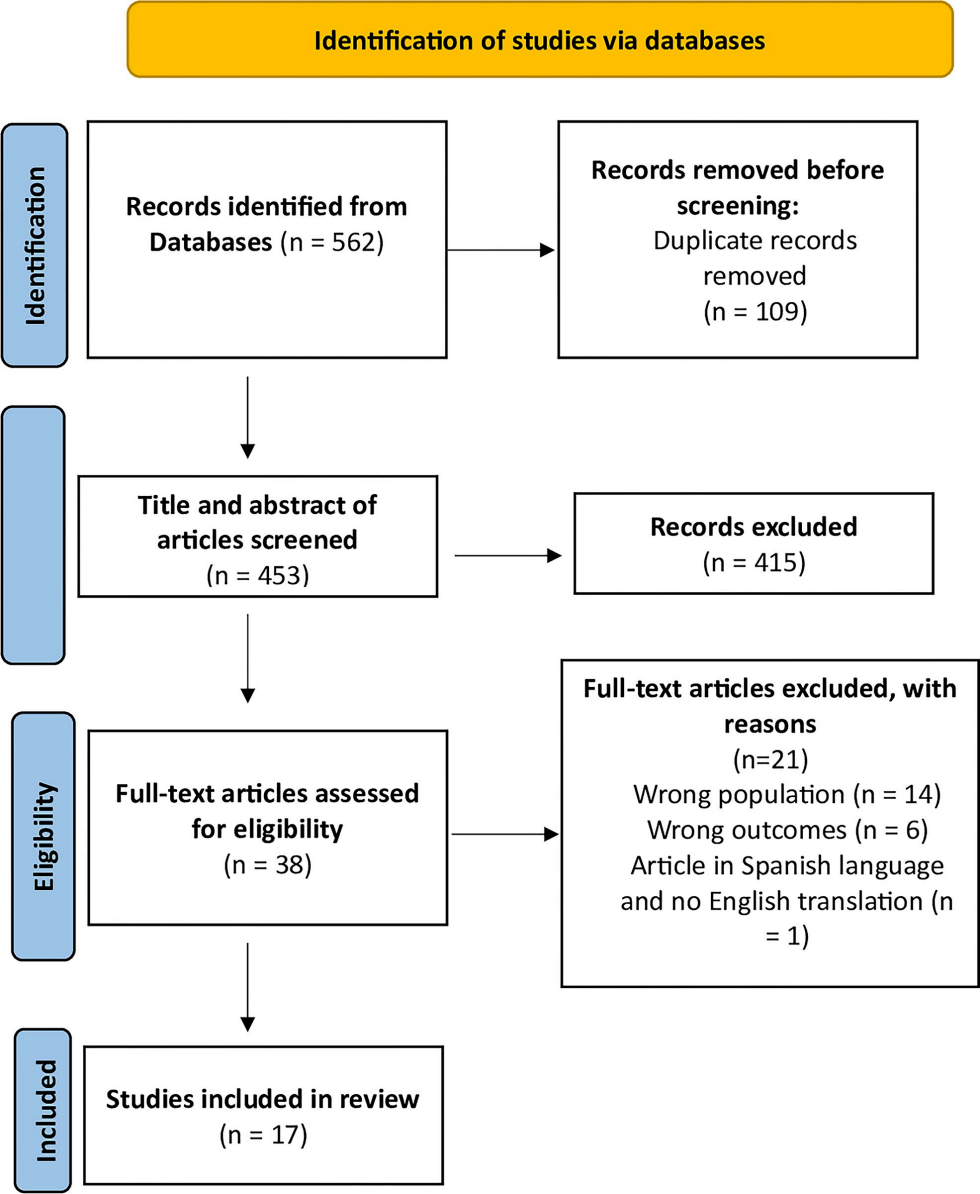
Systematic review PRISMA flow diagram.

All authors independently reviewed articles to identify relevant studies by following the inclusion and exclusion criteria described in Table 2. All articles were imported into Covidence, an online tool designed for managing systematic reviews [20]. Duplicates were identified and removed. The articles underwent a three-step selection process (see Fig 1). Following the removal of duplicates, articles underwent title and abstract screening. Any article that did not meet the inclusion criteria was removed, while those that met the inclusion criteria were retained, followed by a full-text screening of articles that met the inclusion criteria. The articles were screened at the full-text stage for eligibility, all authors independently read the articles at this stage to determine if the remaining articles met the inclusion criteria. Articles that did not meet the inclusion criteria at this stage were removed. During each step, any disagreements concerning eligibility were resolved through discussion between authors to reach a consensus.

**Table 2:** Inclusion and exclusion criteria used for abstract and full-text screening.

Inclusion Criteria	Exclusion Criteria
1. All study types (irrespective of study design). 2. Studies on migrant pregnant and postpartum women from low-and middle-income countries. 3. Studies conducted in high-income countries, as defined by World Bank as those with a Gross National Income (GNI) per capita of $13, 205 or more in 2021 (World Bank, 2023). 4. Studies that include pregnant women who have migrated from low- and middle-income countries to live in a high-income country. 5. Main outcomes were cultural food practices and sources of nutrition information during pregnancy and postpartum in high income countries. 6. Studies published in English language. 7. No date restrictions take place.	1. Conference proceedings and conference abstracts. 2. Non-immigrant pregnant or postpartum women from low-and middle-income countries. 3. Studies conducted in low-and middle-income countries. 4. Studies that do not include pregnant or postpartum women from low- and middle-income countries. 5. Systematic reviews and reviews (including meta‐analyses, narrative reviews, and scoping reviews). 6. Main outcomes were not cultural food practices or sources of nutrition information during pregnancy and postpartum in high income countries.

### Data extraction

Data were extracted into Microsoft Excel. The corresponding author extracted all data, the second and third authors reviewed and cross-checked the extraction. Data, including key characteristics, participants’ country of origin, destination country, age, level of education, employment status, years of migration, gestational age, common food practices during pregnancy and postpartum, reasons for such practices, the source of information from which pregnant women draw their nutrition information, data collection, and any other key findings, were extracted from all articles.

### Quality assessment

By critically evaluating each study’s methodological quality, including how much it addressed the possibility of bias in its design, conduct, and analysis, the quality of all extracted studies was determined. This assessment was conducted using the Joanna Briggs Institute’s (JBI) critical appraisal tools, which were found effective as used in other systematic reviews [21–23]. JBI has a separate appraisal tool for each study design. The tools were used according to the types of study designs used in each of the extracted articles [24, 25].

A rating of “yes”, “no”, “unclear”, or “not applicable” was assigned to qualitative studies based on the answers to 10 validity questions. The same rating scale was applied to cross-sectional studies, which had 8 validity questions, and to cohort studies, which had 11 validity questions.

Consistent with Shi and colleagues [23], the quality of each study was rated as “good” (only yes or not applicable ratings), “fair” (1 to 2 no or unclear ratings), or “poor” (3 or more no or unclear ratings), depending on the number of affirmative responses to the validity questions. The quality of the qualitative studies was assessed using the 10-item qualitative critical appraisal checklist [24]. This checklist evaluated 10 factors within the study, including the congruity between the research methodology and the philosophical perspective, research questions or objectives, data collection, data analysis, results interpretation, influence of the researcher, representation of the participants, ethical approval, and the flow of the conclusion. The cross-sectional studies were assessed for eight factors, including the definition of the inclusion criteria for the sample, the description of the subjects and setting, the measurement of exposures, conditions, and outcomes, the identification and strategies to address confounding factors, and the appropriateness of the statistical analysis method [25]. For the cohort studies, assessment criteria included changes over time, the sufficiency of the follow-up period, loss to follow-up, and the appropriateness of strategies to address loss to follow-up [25].

### Data synthesis

Data were synthesized following a narrative reporting method [22, 26], with information summarized and presented descriptively. The descriptive analysis involved extracting relevant information from each study. No quantitative synthesis, such as a meta-analysis, was conducted due to the heterogeneity in study outcomes among the included quantitative studies. The quantitative data synthesis involved a comprehensive extraction and analysis of numerical data reported in the included studies. The narrative reporting method was followed, where quantitative findings were extracted from each study and synthesized descriptively. This process included identifying key numerical results, such as means, ranges, percentages, and summarizing them in the narrative synthesis. This review adheres to the PRISMA (Preferred Reporting Items for Systematic Review and Meta-Analysis) Statement [27].

## Results

The database search generated 562 articles, of which 109 were duplicates. The titles and abstracts of 453 articles were screened, and 415 articles were excluded because they did not refer to or explore the cultural food practices and sources of nutrition information of pregnant or postpartum migrants from LMICs. The remaining 38 articles were included in full-text review, with 21 articles excluded as they did not meet the inclusion criteria. This resulted in 17 articles remaining for inclusion in this review (Fig 1).

### Description of included studies

17 studies were included in this review: ten qualitative studies [28–37] and seven quantitative studies (four cross-sectional and three cohort studies) [38–44]. Articles were published between 1987 and 2024. The study populations included pregnant migrant women (n = 7) [32, 34–36, 38, 40, 41], postpartum migrant women (n = 7) [28–31, 37, 43, 44], and both pregnant and postpartum migrant women (n = 3) [33, 39, 42]. The total number of participants in all the studies was 2,415 (participant numbers ranged from 10 to 26 in qualitative studies and from 38 to 1,027 in quantitative studies).

Participants originated from Asian (n = 13) and African countries (n = 4). Their destination countries included Canada (n = 6) [30–34, 44], the USA (n = 4) [37, 40–42], Sweden (n = 2) [28, 29], Singapore (n = 2) [39, 43], the UK (n = 2) [35, 36], and Ireland (n = 1) [38]. Ten studies [28, 30, 32–34, 37, 38, 40–42] reported the duration of time the migrant women had lived in high-income countries. The mean years of migration to the destination countries ranged from 2 to 12 years (range 0 to 20 years). Four studies [34, 38, 39, 41] reported participants’ gestational age, which ranged from 14 to 38 weeks. Refer to Table 3 for detailed information.

**Table 3:** Characteristics of the included studies.

Author & year	Settings (Country research undertaken in)	Study design	Study population	Sample size	Sampling	Country of origin	Age (years)	Years of migration	Level of education	Employment status	Gestational age (weeks)	Parity	Data collection	Sociodemographic factors
Ahlqvist & Wirfält (2000) [28]	Sweden	Qualitative	Postpartum women	14	Purposeful and network	Iran	Mean = 45 Range = 29 to 85	Range = 2 to 20	University degree (14%), Primary education (14%)	Not Reported	Not Reported	Range = 2 to 4	Open-ended and focused interviews	Social group
Chen et al. (2014) [39]	Singapore (Women’s and Children’s Hospital and National University Hospital)	Quantitative (Cohort)	Pregnant and postpartum women	1027	Clinical recruitment	China, Malay, and India	Mean = 30.4 Range = 18 to 50	Not Reported	Primary education (4.2%), Secondary education (36.2%), Pre-university (24.3%), University degree (32.9%).	Not Reported	Range = 26^th^ to 28^th^	Not Reported	Interviewer administered questionnaire, 24h recall, and home visit.	Not Reported
Dennis et al. (2024) [44]	Canada (Toronto, Ontario)	Quantitative (cohort)	Postpartum women	493	Referrals and self-referrals from public health visitors, community organizations, study flyers, and ethnic newspapers	China	Not Reported	Not Reported	92% had post-secondary education	Not Reported	Not Reported	Not Reported	Data were collected through a telephone survey	Not Reported
Essén et al. (2000) [29]	Sweden	Qualitative	Postpartum women	15	Opening meetings held by authors	Somalia	Range = 20 to 55	Not Reported	Not Reported	Not Reported	Not Reported	Rage = 2 to 9	In-depth interviews	Level of education
Grewal et al. (2008) [30]	Canada (large urban centre in British Columbia)	Qualitative	Postpartum women	20	Purposeful and network	India	Mean = 25.6 Range = 21 to 30	Mean = 2 Range = 0 to 5	Not Reported	Paid labour (40%)	Not Reported	Not Reported	Face-to-face interviews and focus group discussion with healthcare professionals and community leaders	Social group
Groleau et al. (2006) [31]	Canada (Mid-size city in Québec)	Qualitative	Postpartum women	19	Purposive and snowball	Vietnam	Between23 and >40	Not Reported	Primary education 47.4%), High school (10.5%), College/University degree (37%))	Not Reported	Not Reported	Range = 1 to 6	Face-to-face interviews	Not Reported
Higginbottom et al. (2014) [32]	Canada (Prenatal and Obstetric Clinic in Edmonton)	Qualitative	Pregnant women	10	Purposive sampling	Africa and South Asia	Mean = 33.5	Range = 2 to 4	Not Reported	Employed (30%)	Not Reported	Mean = 1.6	Photo voice and semi-structured photo-elicited interviews	Level of education, parity
Higginbottom et al. (2018) [33]	Canada	Qualitative	Pregnant and postpartum women	23	Purposive sampling	China	Mean = 31.6 Range = 26 to 42	Range = 0 to 10	All completed some form of post-secondary education	Not Reported	Not Reported	Not Reported	Photo voice and semi-structured photo-elicited interviews	Level of education, socioeconomic status
Hussain et al. (2021) [36]	UK (Local community centre)	Qualitative	Pregnant women	10	Convenience sampling	Pakistan	Range = 30 to 40	Not Reported	Not Reported	Not Reported	Not Reported	Not Reported	Semi-structured interviews were conducted in the presence of female Pakistani community link worker.	Social group
Karki et al. (2022) [40]	USA (community centres in Utah)	Quantitative (Cross-sectional)	Pregnant women	38	Convenience and snowball sampling	Bhutan	Mean = 29.6 Range = 21 to 37	Range = 3 to 6	High school (61%), College education (22%), No formal education (11%)	Not Reported	Not Reported	Not Reported	A structured survey	Level of education
Legault et al. (2014) [34]	Canada (Montreal)	Qualitative	Pregnant women	14	Convenience sampling	North Africa	Mean = 32 Range = 20 to 39	< 5	University degree (79%)	Employed (28.6%), unemployed (64.3%)	15 to 29	Not Reported	In-depth face-to-face interviews	Social group
Lindsay et al. (2014) [38]	Ireland (Antenatal clinics at the National Maternity Hospital)	Quantitative (Cross-sectional)	Pregnant women	52	Recruited by research dietitian	Nigeria	Mean = 32 Range = ≥ 18	Mean = 7.5 Range = <5 to ≥ 10	Not Reported	Not Reported	Mean = 16	Not Reported	Structured questionnaire, dietary intake were assessed by a weekday single 24h recall.	Social group
Lindsay et al. (2021) [41]	USA (Massachusetts)	Quantitative (Cross-sectional)	Pregnant women	86	Snowball sampling	Brazil	Mean = 28.3 Range = 19 to 39	Mean = 10.7	Completing high school or less (35%)	Not Reported	Mean = 27.5 Range = 14 to 38	Not Reported	Interviewer-administered surveys, either in person or via telephone, in the participants’ preferred language.	Not Reported
Qureshi et al. (2013) [37]	USA (New Jersey)	Qualitative	Postpartum women	26	Purposive and Snowball	Pakistan	Mean = 38.5	Mean = 12	High school education (19.2%), Bachelor’s degree (65.4%), Master’s degree (15.4%).	Homemaker (34.6%) Outside employment (65.4%)	Not Reported	Mean = 3	Two interviews were conducted with each participant, involving repeated visits to their homes.	Not Reported
Stewart et al. (1987) [42]	USA (Des Moines and Marshalltown, Central Lowa)	Quantitative (Cross-sectional)	Pregnant and postpartum women	52	Convenience sampling	Laos	Mean = 40 Range = 21 to 67	Mean = 7	High school (30%)	Blue-collar jobs outside the home (> 50%)	Not Reported	Mean = 5	Structured interview	Level of education, maternal age
Teo et al. (2018) [43]	Singapore	Quantitative (Cohort)	Postpartum women	490	Clinical Recruitment	China and India	Range = 18 to 50	Not Reported	University graduate	Not Reported	Not Reported	Not Reported	Interviewer-administered questionnaires and postnatal dietary intake.	Level of education, maternal age
Yeasmin et al. (2013) [35]	UK (Borough of Tower Hamlets)	Qualitative	Pregnant women	26	Purposive and snowball sampling	Bangladesh	Rang = 15 to 44	Not Reported	Secondary education (46%), Primary education (38%), and University graduates (15%)	Not Reported	Not Reported	Mean = 1	One-to-one interviews and focus groups	Social group

### Sociodemographic factors

Studies reported that sociodemographic factors such as social group [28, 30, 34–36], level of education [29, 32, 33, 40, 42, 43], maternal age [42, 43], parity [32], and socioeconomic status [33] influenced the cultural food practices of pregnant or postpartum migrants in the host countries.

Social groups were identified as one of the influencing factors on the cultural food practices of migrant women during pregnancy in their destination countries. Five studies [28, 30, 34–36] indicate that migrant women were influenced by their friends and senior colleagues who had previously given birth. For instance, in the studies by Yeasmin and colleagues [35] and Hussain and colleagues [36], it was reported that friends and senior colleagues advised migrant women to avoid consuming cucumber during pregnancy because they believed it could cause skin blemishes on the baby after birth. They also advised against consuming large quantities of food during pregnancy, as they believed it could result in difficult delivery.

The level of education among migrant women was another significant factor influencing food practices during pregnancy [29, 32, 33, 40, 42, 43]. Their education level played a crucial role in how these women adhered to their cultural food practices during pregnancy or postpartum. Two studies [42, 43] reported that migrant women who adhered to their cultural food practices during pregnancy or postpartum generally had higher education levels and were older. In contrast, Higginbottom et al. [33] found that the education level of younger women influenced them to choose not to adhere to traditional practices, as they viewed them as no longer relevant or reasonable.

### Cultural food practices

Results relating to cultural food practices during pregnancy and the postpartum period are detailed in Table 4. Fourteen studies (82%) reported that participants maintained their cultural food practices during pregnancy and postpartum in their destination countries [28–33, 35, 36, 38–40, 42, 43]. Three studies [37, 38, 41] reported limited adherence to cultural food practices during pregnancy and the postpartum period. Limited adherence was due to a lack of support from family members in the host country, food acculturation which led migrant women to discover new food products, and lack of access to traditional foods in the host country. One study [37] reported that many participants longed for their traditional foods in the postpartum period but could not have them due to a lack of support from family members. It was further reported that migrant women attributed their subsequent poor health to the omission of traditional foods [37].

**Table 4:** Cultural food practices, sources of nutrition information and hinderances to cultural food practices.

Author & year	Cultural Food Practices				Sources of Nutrition Information	Hindrances to Cultural Food Practices	Summary of Findings
	Pregnancy food practices	Reasons for pregnancy food practices	Postpartum food practices	Reasons for postpartum food practices			
Ahlqvist & Wirfält (2000) [28]	The consumption of fish, sour foods (e.g., cherry), fat, and protein foods during pregnancy. Avoided the consumption of carbohydrate foods (rice, bread, potatoes) for some weeks towards the end of pregnancy.	**Benefit for the child:** Increase the beauty, mental capacity, lower birth weight, and stamina of the child, including both physical strength and resistance to disease, as well as affecting the child’s future food preferences. **Benefit to the mother:** It provides strength, aids in pushing during delivery, and ultimately facilitates survival.	Their postpartum diet consists of butter or oil, sugar, wheat flour, almonds, nuts, and/or saffron.	It is believed that these foods are hot and good for the womb, beneficial for stomach pain and aiding digestion, reducing bleeding, promoting the production of breast milk, and restoring a woman’s capacity to work after delivery.	Mothers, grandmothers, and mothers-in-law.	The availability of their cultural food is low, and the prices are high (expensive).	Foods were thought to maintain a ’hot-cold’ balance, boosting well-being for both mother and breastfed child. The diet before delivery provided strength for birth. The postpartum diet compensated for lost strength, aiding maternal health, work, and breastfeeding. Valued experiences of mothers and grandmothers. Migration led to altered food choices due to cost, reducing the intake of nutritious items like green leafy vegetables rich in iron, folic acid, and carotene.
Chen et al. (2014) [39]	**Foods increased during pregnancy:** milk, fruit, vegetables, and rice, noodles and bread, fish. **Foods Decreased during Pregnancy:** tea, eggs, coffee, soft drinks, seafood, and confectionery.	Not Reported.	**Foods increased during postpartum:** fish, leafy vegetables,milk-based drinks, ginger, and garlic consumption. **Foods Decreased during Postpartum:** chocolates, sweets, noodles, seafood, cheese, yoghurt, fruit juice, soft drinks, eggs, and beef.	Not Reported.	Not Reported.	Not Reported.	Singaporean women changed their food consumption during pregnancy and postpartum, blending modern dietary advice with traditional beliefs. During pregnancy, 39.2% to 66.9% of participants increased their food intake. Ethnic disparities emerged, notably in egg consumption, with Malays decreasing intake more (28.6%) compared to Chinese (11.1%) and Indian (16.9%) participants. Postpartum, 47.1% to 73.4% increased fish, leafy vegetables, and milk drinks. Ethnic differences persisted, such as Chinese participants favouring ginger and wine/alcohol in cooking, and Indians consuming more milk and garlic. Malays showed varied changes, including decreased egg and beef consumption for half of the participants.
Dennis et al. (2024) [44]	Not Reported	Not Reported	**Food consumed:** nutritious Chinese foods and soups. **Foods Avoided:** Cold foods, cold drinks, spicy foods, and raw food	To increase their milk supply, participants consumed soup.	Not reported	Not reported	The participants consumed only specific types of food, with dietary practices being the most prevalent rituals influenced by Chinese culture. Among the participants, 71.9% were women who adhered to these dietary rituals, attributing them to Chinese cultural traditions. 75.6% women practiced postpartum ritual for a duration of 30 days, motivated by both tradition and the belief in their health benefits (69.1%). They believed these practices would enhance their current (81.9%) and future (83.4%) health, while 64.2% perceived benefits for their baby’s health. However, 29.3% of women indicated feeling pressured or obligated to engage in these rituals.
Essén et al. (2000) [29]	Reduction of food intake during pregnancy.	To avoid having a large fetus and the complications it might bring during labour.	Not Reported.	Not Reported.	Not Reported.	Not Reported.	The participants reduced their food intake during pregnancy, possibly with the intention of avoiding difficulties during delivery, including cesarean sections.
Grewal et al. (2008) [30]	Fresh fruits, vegetables, and milk. Also include foods that they craved, especially ones that were common “back home,” such as corn roti (flatbread), mangoes, and saag (curried spinach and mustard leaves). Late in pregnancy, they ate Soonf (fennel seeds) roasted in brown sugar.	To prevent deficiency of nutrients during pregnancy. Soonf (fennel seeds) roasted in brown sugar is consumed to encourage the onset of delivery or induce pains (during labour).	Dahl (lentil soup), khicheri (lentil soup with rice), roti (flatbread), chai (fennel seed tea with ginger), ginger curry, panjiri, chuanan, and dabrha, which were made from “heat-producing” ingredients such as nuts, raisins, dates, ginger powder, fennel seeds, raw sugar, flour, ghee, and special herbs.	These types of food are seen as essential for healing and recovery from the birthing process, including relieving back pain, promoting menstrual flow to cleanse the body, building the mother’s milk supply, and preventing weakness and illness in later life.	Family members, relatives, husbands, and female elders including mothers and mothers-in-law.	Not Reported.	Participants expressed the importance of food being served hot during the postpartum period because they believed that cold food could disrupt the mother’s body balance after childbirth. The primary sources of information during pregnancy were female family members. Health professionals reported that new mothers within the Punjabi community are expressing concerns about the high fat content of many traditional foods. They are requesting their families to prepare "low-fat" versions of these foods.
Groleau et al. (2006) [31]	Not Reported.	Not Reported.	They engaged in the consumption of hot foods during the postpartum period, and these included strong spices like salt, pepper mixed with hot peppers, bitter juice, pork ragout, and green papaya.	To restore lost blood and heat during delivery and regain strength and equilibrium necessary for health, as well as to produce fresh and nourishing milk.	Not Reported.	Absence of postpartum support from key family members in the host country.	The belief in the consumption of hot foods during the postpartum period was evident among these participants. Not all the participants were able to engage in their traditional nutrition practices during the postpartum period due to the absence of key family members in the host country.
Higginbottom et al. (2014) [32]	Increased the intake of butter in every meal, drinking liquids, milk, and apples.	To improve ease of delivery; the consumption of milk and apples is to give birth to a fair-skinned baby.	Sweets, nuts, hot tea with cinnamon, cinnamon with ginger, more protein foods, nuts, and palm fufu with soup. **Foods avoided:** Starchy foods (rice, pitas), spicy foods, junk food, fatty food, alcohol, and coffee.	To replenish lost energy during delivery, cleanse the blood after delivery, and help produce more milk for breastfeeding.	Family members, friends, biomedical health care providers, community members, and media.	Geographical location and some foods were simply not available or too expensive.	Participants favoured advice from family members despite having lived in Canada for ten years. There was conflict between the advice they got back from home and the healthcare professional’s advice. The traditional food practices of hot and cold foods were also evident in this study.
Higginbottom et al. (2018) [33]	Increased the consumption of milk and milk products, fruits and vegetables, meat, eggs, tofu, soup, congee (a thick porridge of rice), Bird saliva, white fungus, fish, nuts, and black sesame. **Foods avoided:** Deep-fried foods, fast foods, junk foods, coffee, tea, and other caffeinated beverages, raw meat and fish, consuming and cooking with alcohol, job’s tears seeds, black fungus, rabbit meat, snake meat, lamb meat, fruits that are red or orange in colour, spicy foods, and ice cream.	**Eggs and soup:** Baby born with light-coloured and flawless skin (a highly desirable physical attribute in Chinese culture). **Bird saliva (specifically swallow saliva), white fungus:** Beautifying effects. **Increased fish intakes:** Aid in brain development. **Nuts and black sesame:** Aid in brain development and produce thick black hair. Foods were avoided because they were believed to be unhealthy in general, would cause miscarriage, would inhibit lactation, or would have a negative influence on their child’s health and appearance.	Soup (prepared with vegetables, fish, meat, and/or bones), daylily flowers, black fungus, ginger, and limit intake of certain fruits and vegetables (hot), ginger, deep-fried foods, foods that are black in colour (such as black vinegar, black sugar, and Silkie chicken (which has black skin and bones and greyish-black meat)). **Foods Avoided:** salty foods, seafood (especially crab), certain fruits (watermelon, pineapple, and honeydew), and herbal tea.	They are restorative foods that provide energy, promote healing, help balance the body, help the uterus contract, and aid them in shedding ‘bad blood.’ Foods were avoided because they believed they could harm the baby, decrease lactation, and promote bleeding.	Health practitioners, family members, friends, relatives, partners, community members, and media sources. Some women received unsolicited advice from people in their social environment, while others sought out advice from more experienced women.	Lack of social support, changes in their health beliefs, lifestyles of the women and the time available for food preparation.	Participants practiced traditional food categorization as hot or cold based on perceived effects, not physical temperature, for humoral balance during the perinatal period. They got advice from relatives, especially mothers, and health workers.
Hussain et al. (2021) [36]	Cold foods such as apples and grapes were consumed in the first and second trimesters and avoided in the third trimester. Hot foods such as fish, eggs, meat, nuts, and some fruits (like mango and dates) were consumed in the third trimester and were avoided in the first and second trimesters. However, hot foods were consumed before one month of the pregnancy. The participants also engaged in the reduction of food intake.	Cold foods are consumed in the first and second trimesters to provide a cooling effect to the body. However, it was avoided during the third trimester to prevent muscle expansion and painful delivery. Hot foods are consumed in the third trimester for easy delivery. Hot foods were avoided during the first and second trimesters to prevent increased body heat and miscarriage.	Not Reported.	Not Reported.	Family members and in-laws, particularly the woman’s mother-in-law.	Not Reported.	The participants categorized the food they consumed during pregnancy as either ’hot’ or ’cold’ based on their perceived effects on pregnant women’s bodies. They reduced their food intake during pregnancy with the aim of preventing a difficult delivery and reducing the baby’s size.
Karki et al. (2022) [40]	72% consumed dairy 1–2 times per day, 72% consumed beans or lentils 1–2 times per day, and 80% ate fruit 1–3 times per day. However, meat consumption was low as most (81%) ate little or no meat.	Not Reported.	Not Reported.	Not Reported.	Health workers (76%) and female family members (53%).	Not Reported.	Participants followed traditional dietary habits upon resettlement and were able to access culturally acceptable food. However, most participants in the study had spent three to six years in the US, which may have played a role in their ability to easily navigate culturally acceptable food.
Legault et al. (2014) [34]	The preparation of traditional dishes and the use of Maghrebian spices. **Avoided:** spicy foods and cinnamon.	To protect the baby from having health problems, and to prevent miscarriage.	Not Reported.	Not Reported.	Social networks, healthcare providers (physicians, nurses, and registered dietitians), books, and the internet.	Not Reported.	Participants had limited adherence to cultural food practices. Some women experienced food acculturations, which led them to discover new food products. 29% of the participants reported that eating habits acquired from their home country prompted them to seek information in books, on the internet, or from their social network. 21% of the participants perceived that family members, such as their husbands, lacked the expertise to provide nutrition information.
Lindsay et al. (2014) [38]	Rice and other grains, meat, fish, vegetables, tubers, and oils, the traditional Nigerian stew (a spicy tomato-based stew containing vegetables (such as onion, chili pepper, okra, and green leafy vegetables), pounded or ground yam, cassava, homemade soups (vegetable-based, a few pieces of meat or fish), and egusi soup.	Not Reported.	Not Reported.	Not Reported.	Not Reported.	Not Reported.	Traditional African dishes were consumed by almost all participants, despite the majority having lived in Ireland for several years. 80% of the participants frequently consumed starchy carbohydrate foods such as grains, rice, pasta, and savory items. The participants also had regular consumption of the following food groups: "vegetables and vegetable dishes (98%)", "meat and meat dishes (95%)", "milk and yogurt (95%)", and "butter, spreading fats, and oils (89%)".
Lindsay et al. (2021) [41]	Not Reported.	Not Reported.	Not Reported.	Not Reported.	Internet (81·3%), family and friends (71%), and Healthcare providers (81.4%).	Not Reported.	About three quarters (72·1%–81·3%) of respondents turned to the internet for information about diet during pregnancy while 71% of the participants sought advice from family members and friends. 81.4% sought advice from healthcare providers.
Qureshi et al. (2013) [37]	Not Reported.	Not Reported.	**Panjeeri:** A mixture containing semolina, butter, nuts, seeds, and sugar; **Kaara:** Warm-sweetened milk with butter and turmeric and/ or ground nuts, and **Yakhni:** A clear soup made of chicken, goat, or beef.	They believed that these foods have a fortifying power and hasten healing after delivery.	Not Reported.	Lack of social support.	Participants were unable to follow traditions that they believed promoted their health and that of their babies. They attributed their consequent poor health to this omission. These were especially difficult when they had no family members to guide them and perform the rituals. Many mothers longed for the traditional foods in postpartum.
Stewart et al. (1987) [42]	Increased meat consumption (especially pork and chicken), fruits and vegetables, consumption of less foods.	To give energy, ease the strain of delivery, prevent nausea, and diarrhea.	**Foods consumed:** Tea and hot water for one month, ate only boiled foods for one month, increased consumption of chicken, pork, and vegetables. **Foods Avoided:** Hot spices and alcohol.	To restore the mother’s strength after delivery. Foods were avoided to prevent diarrhea after delivery.	Not Reported.	Not Reported.	The women advocated for increased consumption of tonic foods such as chicken, fruits, vegetables, and pork, while decreasing or avoiding alcohol and hot spices during pregnancy. However, 65% advocate for an increase in the quantity of food intake, while 35% advocate for a reduction in food quantity during pregnancy.
Teo et al. (2018) [43]	Not Reported.	Not Reported.	Consumption of dried fruits, herbal tea, rhizomes, foods cooked with wine, alcohol, or vinegar (which are considered "hot" foods), ethnic bread, whole milk, butter/ghee, garlic, deep-fried/mashed potatoes, sweetened and carbonated drinks, ice cream, chips/crisps, local savory snacks, high intakes of assorted soup (vegetables, meat, fish, seafood, and noodles), vegetables, non-fried fish, and fresh fruits.	Not Reported.	Not Reported.	Not Reported.	The participants consumed their traditional foods during the postpartum period, as well as incorporating western diets, which included foods from the host country. Four dietary patterns were followed during the confinement period, and these are Traditional-Chinese- Confinement (TCC), Traditional-Indian-Confinement (TIC), Eat-Out diet, and Soup-Vegetables-Fruits (SVF).
Yeasmin et al. (2013) [35]	Participants categorized foods as "good" and "bad" based on their beliefs about certain foods. Good foods were consumed during pregnancy, while bad foods were avoided or restricted. **Good Foods:** Fruits or fruit juice, vegetables (particularly leafy vegetables), milk, fish, and water. **Bad Foods:** Peanuts, leafy vegetables, goat meat, and lentils. Some foods were restricted during the first trimester (first 3 months) of pregnancy, such as pineapple, papaya, liver, cucumber, duck meat, peanuts, and hot foods.	Major cultural concepts originated from the fear of early miscarriages, particularly in the first trimester. Good foods were primarily consumed for various reasons, such as minimizing constipation and improving the baby’s skin texture (fish). Bad foods were avoided due to reasons like preventing allergies in infants, avoiding rough or spotty skin (cucumber), unpleasant smell, harsh voice (goat meat), and reducing difficulties associated with gas buildup (Lentils).	Not Reported.	Not Reported.	Family members, relatives, and friends	Not Reported.	Cultural influences play a significant role, even for those who have lived in the UK for over a decade. Food intake during pregnancy is heavily influenced by culture, with pregnant women categorizing food as "good" or "bad." However, this classification varies based on individual beliefs and knowledge. What is considered good for one person may be seen as bad for another. Interestingly, many participants are unsure why they avoid certain foods.

Cultural food practices adhered to during pregnancy aimed to ensure successful delivery and healthy outcomes for the baby [28–30, 32–36]. The practices of cold and hot foods beliefs were identified in Ten studies [28, 30–33, 36, 39, 42–44] involving participants who had migrated from Asian countries during pregnancy and the postpartum period. These studies suggest that during pregnancy, a pregnant woman is believed to be in a “hot state” so she should consume cold foods, such as apples and grapes while avoiding hot foods such as spicy foods, ice cream, fish, eggs, and meat [33, 36, 42]. Postpartum is considered a “cold state” and seven studies [28, 30, 31, 33, 39, 42, 43] reported that migrant women engaged in the consumption of hot foods during this period to restore balance to the body after delivery, aid in healing, and facilitate recovery from the birthing process [28, 30]. Hot foods consumed include chicken, pork, certain vegetables (e.g., mustard greens), deep-fried foods, bitter juice, green papaya, hot tea with cinnamon, foods cooked with wine, alcohol, and vinegar, dahl (lentil soup), ginger curry, panjiri, chuanan, dabrha, tea, and hot water for one month, and ate only boiled foods for one month [30, 33, 42, 43]. The comprehensive list of foods classified as either cold or hot during pregnancy and the postpartum period can be found in the **S1 Table**.

A study [35] conducted among Asian migrant women in the UK reported participants engaged in the practices of “good and bad foods” beliefs during pregnancy. “Good foods” were consumed during pregnancy while bad foods were avoided. “Good foods” are those considered beneficial for both the mother and the fetus, typically being nutritionally enriched with vitamins and minerals or are rich in energy (protein or carbohydrates), such as fruits or fruit juice, vegetables (particularly leafy vegetables), milk, and fish. Conversely, “bad foods”’ were believed to be harmful to the fetus and the pregnant mother and may include items such as peanuts, goat meat, pineapple, papaya, liver, cucumber, and lentils (see **S1 Table** for comprehensive lists of good and bad foods).

Migrant women also engage in increased or decreased food intake [28–30, 32, 33, 36, 39, 42] and the restriction or avoidance of certain foods during this period due to their cultural beliefs about these foods [28, 31–33, 35, 42]. These practices aim to facilitate easy delivery, prevent miscarriage, enhance the baby’s skin appearance, and protect the baby’s health. Four studies [28, 29, 36, 42] identified that food intake during pregnancy should be less than when not pregnant, this is to avoid having a large baby, with the risk of delivery by cesarean section [28, 36].

Furthermore, six studies [32–34, 39, 42, 44] have identified that migrant women also engage in certain healthy food practices during pregnancy or postpartum. Some of these practices include avoiding spicy foods, alcohol, coffee, caffeinated beverages (including soda), raw meat, raw fish, and salty foods. Among all these healthy food practices, the avoidance of spicy foods, alcohol, and coffee consumption was most commonly practiced, as evidenced in all the studies.

Five studies [28, 31–33, 37] identified some hindrances to cultural food practices during pregnancy and the postpartum periods among migrant women in host countries. These hinderances include a lack of support, especially from family members [31, 33, 37], low availability of their cultural foods [28, 32], the cost of their cultural foods [28, 32], the geographical locations where traditional food can be purchased [32], changes in their health beliefs and migrant women’s lifestyles [33], and the time available to prepare their cultural foods [33].

### Reasons for cultural food practices

Five reasons that migrant women engage in cultural food practices during pregnancy and the postpartum period were identified (see **S2 Table)**. These are maintaining humoral balance, facilitating easy delivery, the benefits to the fetus, healing, recovery, and lactation, and the impact on the baby’s appearance.

#### Maintaining humoral balance

Nine studies [28, 30–33, 36, 39, 42, 43] reported that the cultural food practices in which migrant women engage during pregnancy or postpartum aim to help maintain hormonal balance. These studies suggest that the body is in a hot state during pregnancy and a cold state during postpartum. Therefore, cold foods are to be consumed during pregnancy, while hot foods are expected to be consumed during the postpartum period.

#### Facilitating easy delivery

Four studies [28, 29, 36, 42] reported that migrant women follow cultural practices of reducing food intake during pregnancy to facilitate easy delivery. These studies suggest that reducing food intake during pregnancy will help prevent a large fetus and reduce the risk of caesarean section. Certain foods are also known to aid in delivery ease, such as adding butter to every meal consumed during pregnancy [32] and the intake of roasted fennel seeds (Soonf) in brown sugar [30].

#### Benefits for the fetus

Five studies [28, 32–35] reported that migrant women engage in cultural food practices during pregnancy to protect the fetus’s health. These practices include the use of Maghrebian spices, avoidance of spicy foods, and cinnamon is believed to protect the fetus from health problems [34]. The consumption of fish, nuts, and black sesame, believed to aid in brain development [33].

#### Healing, recovery, and lactation

Eight studies [28, 30–33, 37, 42, 44] reported that during the postpartum period, migrant women consume certain foods believed to aid in healing after delivery and promote lactation for breastfeeding the newborn. These foods are chicken, palm fufu, soup with vegetables, fish, meat, black vinegar and ginger, butter or oil, sugar, wheat flour, almonds, Panjeeri, Kaara, Yakhni, and nuts.

#### Impact on baby’s appearance

Five studies [28, 32, 33, 35, 36] reported that foods are considered to impact the fetus’s appearance positively or negatively after birth. Foods consumed during pregnancy that are believed to have a positive impact on the baby’s appearance include fish, which is believed to result in a lovely face, light complexion, and healthy skin for the newborn [28], eggs are consumed for light-coloured skin [33], and milk is believed to contribute to a white colour for the child due to milk’s whiteness [36]. Conversely, raw meat, fish, tea, coffee, snake meat, and rabbit meat are avoided as they are believed to cause cleft lip and cleft palate [33], goat meat is thought to cause spots on the baby’s skin [35], and cucumber is believed to cause rough skin [35] are avoided during pregnancy due to their perceived negative effects on the baby’s appearance after birth.

### Sources of nutrition information

The sources of nutrition information encompass a variety of channels (**see Table 4**). These include healthcare providers [32–34, 40, 41], female family members (mothers, grandmothers, mothers-in-law) [28, 30, 32, 33, 35, 36, 40, 41], friends [32, 33, 35, 41], the internet [34, 41], other relatives [30, 33, 35], husbands [30, 33, 35], community members [32, 33], media sources [32, 33], social networks [34], and books [34].

Participants relied on nutritional advice from family members in their home countries. Six studies [28, 30, 32, 33, 35, 36] reported that participants strongly adhered to the advice provided by their mothers or mothers-in-law. This adherence was attributed to their experiences [28, 35] and the trust they had in these family members [32]. Conversely, four studies [33, 35, 36, 44] noted that participants felt obligated to follow the advice from their home country to avoid disobeying or disrespecting their elders and to prevent conflicts.

Four studies [30, 32, 33, 36] described migrant women encountering conflicting nutrition advice from healthcare providers and their families. This conflict arose from disparities between the information provided by healthcare professionals and that from their home countries. Additionally, these women found the advice from healthcare providers to be culturally irrelevant [33], and that this advice created an overwhelming atmosphere of ambiguity and fear for migrant women [36].

### Quality assessment

The analysis of bias risk and quality assessment of the studies was assessed using JBI checklists (see **S3 Table)**. Nine studies [30, 32, 34, 36, 37, 39, 41, 43, 44] were rated “good” because the authors adequately addressed the majority of the validity questions, while seven studies [28, 29, 31, 33, 35, 38, 40] were rated “fair”. The “fair” rating was due to their inadequate addressing of the validity criteria, specifically regarding the researcher’s cultural or theoretical positioning [28, 29, 31, 33, 35], appropriate ethical approval [28, 31, 33], statement of the researcher’s influence [33], inability to state strategies for dealing with confounding factors [38, 40], and unclear statistical analysis [40]. Only one study [42] was rated as “poor” because it failed to adequately address the validity criteria regarding defining the inclusion criteria, inability to identify and state strategies for confounding factors, unclear validity and reliability of the outcomes, and inappropriate statistical analysis.

## Discussion

This systematic review marks the first attempt to investigate the continuation of cultural food practices among migrant population from LMICs residing in high income countries. Despite the importance of pregnancy food practices in clinical practice, few reviews [7, 45] have investigated the continuation of cultural food practices during pregnancy and postpartum. These reviews revealed that women continue with various cultural food practices during these periods. Therefore, this systematic review synthesised the evidence regarding the cultural food practices of pregnant and postpartum migrant women from LMICs residing in high income countries. This review extensively examined the influence of sociodemographic factors on food practices during pregnancy and explored the sources of nutritional information during pregnancy while residing in high income countries.

This review demonstrates that migrant women from LMICs continue to adhere to their cultural and traditional food practices in high income countries during both pregnancy and the postpartum period. Two studies [32, 35] indicated that even after spending more than a decade in their destination countries, women continued to strictly follow their cultural and traditional food practices.

This review identified the categorisation of food as “hot”, “cold”, “good”, and “bad” within the context of cultural food practices, which resonates deeply with the principles of ‘symbolic interactionism theory’. This theoretical framework suggests that individuals’ interactions with objects, including food, are heavily influenced by the meanings they attribute to them, which are shaped by social interactions [46, 47]. These meanings, developed within specific cultural contexts, wield significant influence over individuals’ food decisions, often transcending objective truths [47]. Our findings reveal commonalities in the cultural food practices of migrant women from South Asian countries, particularly concerning beliefs surrounding humoral balance during pregnancy and the postpartum period. Notably, these women adhere to traditional beliefs, consuming “cold foods” during pregnancy and “hot foods” after birth. This finding aligns with a systematic review conducted by de Diego-Cordero and colleagues [7], which highlighted similar food practices among migrant women living in their home countries in LMICs, rooted in the humoral properties of food. The convergence of our findings with existing literature emphasises the profound influence of cultural norms and traditions on food practices during pregnancy within this group of Asian migrant women in high income countries. Moreover, this underscores the importance of cultural sensitivity and awareness when providing healthcare and nutritional guidance to pregnant individuals from diverse cultural backgrounds. Their beliefs and dietary choices may differ significantly from mainstream recommendations.

While some of the cultural food practices can be considered harmless or inconsequential from a health perspective, others may have short or long term implications. Some nutritious foods, such as meat, eggs, and specific fruits (e.g., watermelon, pineapple, apple, papaya, and cucumber), were found to be restricted due to cultural food practices during pregnancy. Such restrictions can have adverse effects, including low birth weight, anaemia, neural tube defects, and premature delivery [48, 49]. These findings are consistent with a recent review by Gebregziabher et al. [22] whereby the authors identified that African migrant women in Africa restrict certain foods during pregnancy, including meat, milk, cheese, certain fruits and vegetables (e.g., banana, cabbage), and legumes. This is problematic because restricting foods like eggs and meat during pregnancy can result in growth retardation and negatively impact the future health of the fetus [22].

Interestingly, this finding reveals that alongside the harmful cultural food practices in which migrant women engage, they also adopt certain healthy practices. For instance, they avoid spicy foods, which can help prevent dyspepsia and heartburn, as well as coffee and alcohol, which can reduce the increased risk of heartburn or other discomfort during pregnancy [50]. These practices align well with the healthy eating guidelines for pregnant women, which recommend limiting or avoiding the intake of spicy foods, coffee, and alcohol during pregnancy [51, 52].

This review identified that sociodemographic factors, such as social group and level of education, significantly influenced the food intake of migrant women from LMICs in their destination countries during pregnancy and in the postpartum period. Women who lived within their cultural groups were more likely to adhere to traditional cultural food practices [30, 35, 36], echoing the findings of D’Souza and colleagues [1], in which migrant women were found to be influenced by the advice from female relatives in their destination country and their community.

Adherence to cultural food practices was more common among older women [42, 43], highlighting a generational difference in food preferences. Conversely, younger women, particularly those with higher levels of education, were less interested in such practices and often consumed less healthy foods, including fast foods and junk food, during pregnancy [33]. This shift raises concerns regarding its potential negative impacts on both maternal and fetal health. To interpret these findings, we turn to the theoretical construct of ‘acculturation’, which elucidates the cultural and psychological transformations that occur when individuals interact with different cultures [53]. In the case of these young women, their shift from traditional food practices aligns with the concept of ‘assimilation’ within the ‘bidimensional acculturation model’. Assimilation, characterised by the rejection of one’s culture of origin in favour of embracing the host culture, emerges as a coping mechanism amidst cultural transitions [54]. This theoretical lens not only contextualises the observed dietary changes but also underscores the significance of cultural adaptation in shaping health behaviours. Consequently, this underscores the need for healthcare providers caring for pregnant migrants in high income countries to educate them on proper dietary intake during pregnancy, considering the dynamics of acculturation and its implications on maternal and fetal well-being.

This review highlights the major sources of nutritional information for migrant women during pregnancy and postpartum in destination countries. The major sources of information were family members [28, 30, 32, 33, 35, 36, 40, 41] and healthcare professionals [32–34, 40, 41]. Notably, nutritional advice from female family members, especially mothers or mothers-in-law from their home countries, was highly valued by migrant women compared to advice from healthcare professionals. However, it was noted that some women received contradictory information from these sources [30, 32, 33, 36], with migrant women often perceiving healthcare professionals’ advice as culturally inappropriate. Similar situations were observed among Kenyan immigrant women in the US, as reported by Chebet [55]. This finding underscores the importance of culturally appropriate nutritional information being made available and accessible to pregnant migrant women in various host countries. Such education should be provided by healthcare professionals to support optimal maternal and child health.

Although most of the studies (n = 13) included in this review focused on migrant women from South Asian countries and only four studies were among African migrant women, it is evident that there is a relative paucity of research conducted among migrant women from LMICs in their destination countries. This paucity of research is consistent with findings from other reviews [1, 7], such as the systematic review by de Diego-Cordero and colleagues [7], where only two of 24 studies were conducted in high income countries (Canada), while the rest of the studies (n = 22) were conducted in migrant women’s home countries (Africa and Asia countries). This underscores the need for more research to better understand whether these women continue to adhere to their cultural food practices in their host countries. Importantly, this review identified a gap in the literature regarding the impact of these cultural and traditional food practices on birth outcomes among migrant women from LMICs in their destination countries. Future research should aim to address these gaps through focused quantitative and qualitative studies.

## Strengths and limitations

This review presents an up-to-date systematic review of the cultural food practices of pregnant migrant LMIC women in their destination countries. The strength of this review lies in the inclusion of various study types, including qualitative, cross-sectional, and cohort studies, without imposing restrictions on publication dates. However, notable limitations must be acknowledged when interpreting our results: only studies published in the English language were included and the search exclusively targeted pregnant and postpartum women. Consequently, articles that identify the food practices of pregnant migrant women in studies conducted with non-pregnant migrant women in their destination countries may be missed.

## Conclusion

The findings in this review demonstrate that pregnant migrant women continue to adhere to their cultural and traditional food practices even after migration. This adherence is strongly influenced by their continued communication with their family members back in their home countries, as well as their relatives, friends, and community members in their destination country. The persistence of these cultural food practices indicates the importance placed on culture by pregnant migrant women. Also, these findings showed that migrant women tend to value nutrition information received from family members and friends back in their home country more than that received from healthcare professionals in the host country because they believe that nutrition information provided by healthcare professionals is not culturally appropriate. Therefore, culturally appropriate nutritional education for pregnant migrant women in various host countries should be available and accessible to women, thereby supporting best maternal and child health. This can be achieved through initiatives such as providing cultural competence training for healthcare professionals, fostering collaboration with community leaders, and organizing community-based events.

The outcomes of this review have the potential to inform the healthcare of pregnant migrant women in various high-income countries. This emphasizes the importance of providing culturally sensitive care and assistance during this critical period. Additionally, these findings can guide policymakers in developing strategies to enhance the food practices of pregnant migrants residing in high-income countries. Future research is needed to explore the research gaps identified in this review, leading to a better understanding of how to support migrant women during pregnancy.

## Supporting information

S1 Checklist PRISMA checklist. (PDF)

S1 Table Comprehensive list of food categories during pregnancy and postpartum and their potential impact on maternal and fetal health. (PDF)

S2 Table Reasons for cultural food practices. (PDF)

S3 Table Quality assessment of studies. (PDF)
