# Supplementary material for: Cultural food practices and sources of nutrition information among pregnant and postpartum migrant women from low- and middle-income countries residing in high income countries: A systematic review
Source: PLoS One. 2024 May 9;19(5):e0303185. doi: 10.1371/journal.pone.0303185 (PMC11081330; doi:10.1371/journal.pone.0303185)
Supplement: S1 Table — (PDF) [file pone.0303185.s002.pdf]

S1 Table. Comprehensive List of Food Categories During Pregnancy and Postpartum and Their Potential Impact on Maternal and Fetal Health.

Authors & Year	Periods	Food Categories	Foods Consumed/Avoided	Potential Impact on Maternal and Baby's Health
Ahlqvist & Wirfält (2000) [1]	Postpartum	Hot Foods	**Consumed:** butter or oil, sugar, wheat flour, almonds, nuts, saffron.	These foods could have a negative impact on the health of the mother and the newborn because they are typically rich in fat and sugar, and are low in essential vitamins and minerals, which could be detrimental to the health of the mother and child.
Grewal et al. (2008) [2]	Postpartum	Hot Foods	**Consumed:** Dahl (lentil soup), khicheri (lentil soup with rice) usually cooked in ghee (clarified butter) and served with roti (flatbread), chai (fennel seed tea with ginger), ginger curry, panjiri, chuanan, and dabrha.	These foods could have a negative impact on the mother and the newborn because it is high in fat.<br>Migrant women in this study expressed concerns about the high-fat content of many traditional foods and are requesting that their families create "low-fat" versions of these foods.
Groleau et al. (2006) [3]	Postpartum	Hot Foods	**Consumed:** Pepper mixed with hot pepper, pork ragout, and green papaya.	The foods could have a positive impact on the mother and newborn's health.
Higginbottom et al. (2018) [4]	Pregnancy	Cold Foods	**Avoided:** Watermelon, pineapple, and honeydew.	These foods avoided during pregnancy could have a negative impact on the mother/fetus's health because they contain essential nutrients (such as vitamins and minerals) that are required for pregnant women, also a good source of antioxidants.
		Hot Foods	**Avoided:** Fruits (red or orange in colour; especially	These foods avoided during pregnancy could have both positive

			lychee), spicy foods, deep-fried foods, sweet foods (like ice cream)	and negative impacts on the mother/fetus's health. The avoidance of fruits could have a negative impact because the fruits contain essential vitamins and minerals that are required for a pregnant woman.
	Postpartum	Hot Foods	**Consumed:** Hot vegetables (e.g., mustard green), chicken fried with alcohol, ginger, deep-fried foods, black vinegar, black sugar, and silkie chicken	These foods consumed during postpartum could have both positive and negative impacts on the mother/newborn's health. For example, deep-fried food could have a negative effect on the mother because it is high in saturated fat and could cause weight gain and other health problems like hypertension.
		Cold Foods	**Avoided:** Seafood (especially crab).	This food avoided could have a positive impact on the baby's health. Some seafood contains high levels of mercury that may be harmful to the central nervous system of the baby.
Hussain et al. (2021) [5]	Pregnancy	Cold Foods	**Consumed:** Apples, grapes	These foods consumed during pregnancy could have a positive impact on the mother or fetus's health because they contain essential vitamins and minerals.
		Hot Foods	**Avoided:** Fish, eggs, meats, nuts, mango, dates	Some of these foods such as eggs, mangos, and meats that were avoided could have a negative impact on the mother's health because these foods contain essential nutrients (vitamins, iron, minerals, and protein) needed for pregnant women.
Stewart et al. (1987) [6]	Postpartum	Hot Foods	**Avoided:** Hot spices, alcohol	The foods could have a positive and negative impact on the

			**Consumed:** Tea	mother and newborn‘s health.
Teo et al. (2018) [7]	Postpartum	Hot Foods	**Consumed:** Dried fruits, herbal tea, rhizomes, and foods cooked with wine, alcohol, or vinegar	The foods could have a positive and negative impact on the mother and newborn‘s health.
Yeasmin et al. (2013) [8]	Pregnancy	Good Foods	**Consumed:** Fruits, leafy vegetables, milk, and fish,	The foods could have a positive impact on the mother and newborn‘s health because they contain essential nutrients (Vitamins, minerals, iron, and protein).
		Bad Foods	**Avoided:** Peanuts, pineapple, papaya, liver, cucumber, duck meat, hot foods, goat meat, and lentils	These foods that were avoided could have a negative impact on the mother’s health during pregnancy because they contain essential nutrients (vitamins, iron, minerals, and protein) required for pregnant women.

Reference

1. Ahlqvist M, Wirfält E. Beliefs concerning dietary practices during pregnancy and lactation. A qualitative study among Iranian women residing in Sweden. Scandinavian journal of caring sciences. 2000;14(2):105-11. PubMed PMID: 12035273.
2. Grewal SK, Bhagat R, Balneaves LG. Perinatal beliefs and practices of immigrant Punjabi women living in Canada. Journal of obstetric, gynecologic, and neonatal nursing : JOGNN. 2008;37(3):290-300. doi: 10.1111/j.1552-6909.2008.00234.x. PubMed PMID: 18507600.
3. Groleau D, Soulière M, Kirmayer LJ. Breastfeeding and the cultural configuration of social space among Vietnamese immigrant woman. Health & place. 2006;12(4):516-26. doi: 10.1016/j.healthplace.2005.08.003. PubMed PMID: 16157504.
4. Higginbottom GMA, Vallianatos H, Shankar J, Safipour J, Davey C. Immigrant women's food choices in pregnancy: perspectives from women of Chinese origin in Canada. Ethnicity & health. 2018;23(5):521-41. doi: 10.1080/13557858.2017.1281384. PubMed PMID: 28158953.
5. Hussain B, Bardi JN, Fatima T. Pregnancy related cultural food practices among Pakistani women in the UK: a qualitative study. British Journal of Midwifery. 2021;29(7):402-9. doi: 10.12968/bjom.2021.29.7.402. PubMed PMID: 151268444. Language: English. Entry Date: 20210709. Revision Date: 20210712. Publication Type: Article.

6. Stewart MM, Whiteford MB. Dietary habits and obstetrical service utilization during pregnancy and lactation among Tai Dam women of central Iowa. Ecology of Food and Nutrition. 1987;20(2):121-42. doi: 10.1080/03670244.1987.9990993. PubMed PMID: 19881405938. Stewart, M. M. (author).
7. Teo C, Chia A, Colega MT, Chen L, Fok D, Pang W, et al. Prospective associations of maternal dietary patterns and postpartum mental health in a multi-ethnic Asian cohort: the Growing up in Singapore towards Healthy Outcomes (GUSTO) Study. Nutrients. 2018;10(3):299. doi: 10.3390/nu10030299 https://www.mdpi.com/2072-6643/10/3/299/htm. PubMed PMID: 20183264536. Teo, C. (author).
8. Yeasmin SF, Regmi K. A qualitative study on the food habits and related beliefs of pregnant British Bangladeshis. Health care for women international. 2013;34(5):395-415. doi: 10.1080/07399332.2012.740111. PubMed PMID: 23550950.
