# Supplementary material for: Cultural food practices and sources of nutrition information among pregnant and postpartum migrant women from low- and middle-income countries residing in high income countries: A systematic review
Source: PLoS One. 2024 May 9;19(5):e0303185. doi: 10.1371/journal.pone.0303185 (PMC11081330; doi:10.1371/journal.pone.0303185)
Supplement: S2 Table — (PDF) [file pone.0303185.s003.pdf]

S2 Table. Reasons for Cultural Food Practices

Groups	Reasons	List of Foods	Effect	References
Baby	Benefits for the fetus	**Consumed:** fish, nuts, and black sesame.<br>**Consumed:** Milk, fruits, and vegetables	To aid in brain development and produce black thick hair<br>To make the baby grow bigger and healthy	[1-3]
		**Avoided:** Maghrebian spices, spicy foods	To protect the fetus from health problems	[4]
		Avoided the consumption of Peanuts and lentils	To prevent allergy for the baby and reduce any difficulties with gas build-up	[5]
	Impact on baby's appearance	**Consumed:** Milk, fruit or fruit juice, eggs and soup, bird saliva, white fungus, apples	Makes the skin colour of the baby white/light-coloured, smoother, nicer, flawless skin, beautifying effects	[1-3, 5, 6]
		**Avoided:** Cucumber, goat meat, duck meat, snake meat, lamb meat, watermelon, pineapple, honeydew<br>**Avoided:** Rabbit meat	Makes the baby's skin rough and smell, to prevent the baby from having a harsh voice, and epilepsy.<br>To prevent baby from having cleft lip and cleft palate	[3, 5]<br>[3]

Mother	Maintaining Humoral balance	**Consumed:** Dahl (lentil soup), khicheri (lentil soup with rice)<br>usually cooked in ghee (clarified butter) and served with roti (flatbread), chai (fennel seed tea with ginger), ginger curry, panjiri, chuanan, dabrha, consumption of tea and hot water for one month, only boiled foods, dried fruits, herbal tea, rhizomes, foods cooked with wine/alcohol/vinegar during postpartum.<br>**Avoided:** The consumption of fruits (red or orange colour), spicy foods, deep-fried foods, and ice cream during pregnancy.<br>**Consumed:** fish, eggs, meat, nuts, and some fruits like mango and dates.<br>**Consumed**: Apples and grapes	Brings the body to balance during postpartum because the body is believed to be in a cold state during this period.<br>These foods are known to be hot, and they were avoided because the body is believed to already be in a hot state during pregnancy.<br>To produce heat in the body and increase the body temperature.<br>To give coolness to the body.	[1-3, 6-11]
	Benefit for mother's health	**Consumed:** cherries<br>**Consumed:** watermelon<br>**Consumed:** Fruits and vegetables<br>**Consumed:** Pork, beef, and chicken, congee (a thick porridge of rice)	To give mother good/clean blood<br>To increase blood in a pregnant woman's body<br>To make the mother healthy<br>For successful pregnancy and also gives energy	[1, 3, 10]

		**Avoided:** Lentils<br><br>**Avoided:** hot spices during pregnancy	To prevent any difficulties in gas build-up<br><br>To prevent nausea and diarrhea	[5]<br><br>[10]
	Miscarriage	**Avoided:** Pineapple, papaya, tamarind, Cinnamon, deep-fried foods, job's tear seeds, black fungus	To prevent the loss of the fetus	[3-5]
	Facilitating easy delivery	**Avoided:** Consumption of carbohydrates (such as rice, bread, and potatoes for some weeks towards the end of pregnancy)<br>Reduced food intakes	To lower the birth weight.	[1, 6, 10, 12]
		**Consumed:** Soonf (fennel seeds) roasted in brown sugar, addition of butter to every meals	To assist in sharpening[inducing] the labour pains	[2, 8]
	Healing, Recovery, and Lactation	**Consumed:** butter or oil, sugar, wheat flour, almonds, nuts, saffron, dahl (lentil soup), chai (fennel seed tea with ginger), ginger curry, panjiri, chuanan, dabrha, soup (prepared with vegetables, fish, meat, and/or bones), daylily flowers, black fungus, black vinegar, Silkie chicken (which has black skin and bones and	Restore the reproductive organs and promote lactation, to reduce bleeding, gives the mother strength.	[1-3, 5, 8-10, 13]

		greyish-black meat), hot tea with cinnamon, cinnamon with ginger, palm fufu, chicken, pork/pork ragout, vegetable, green papaya, yakhni (clear soup), and kaara (a drink of warm sweetened milk with butter and turmeric)		
		**Avoided:** Garlic, cabbage, and onion, starchy foods (rice and pitas), coffee, spicy food	It makes the breastmilk to develop a bad odor, makes the baby gassy and uncomfortable	[1, 2]

Reference

1. Ahlqvist M, Wirfält E. Beliefs concerning dietary practices during pregnancy and lactation. A qualitative study among Iranian women residing in Sweden. Scandinavian journal of caring sciences. 2000;14(2):105-11. PubMed PMID: 12035273.
2. Higginbottom GMA, Vallianatos H, Forgeron J, Gibbons D, Mamede F, Barolia R. Food choices and practices during pregnancy of immigrant women with high-risk pregnancies in Canada: a pilot study. BMC pregnancy and childbirth. 2014;14:370. doi: 10.1186/s12884-014-0370-6. PubMed PMID: 25467067.
3. Higginbottom GMA, Vallianatos H, Shankar J, Safipour J, Davey C. Immigrant women's food choices in pregnancy: perspectives from women of Chinese origin in Canada. Ethnicity & health. 2018;23(5):521-41. doi: 10.1080/13557858.2017.1281384. PubMed PMID: 28158953.
4. Legault A, Marquis M. Nutrition information-seeking behaviour of low-income pregnant Maghrebian women. Canadian journal of dietetic practice and research : a publication of Dietitians of Canada = Revue canadienne de la pratique et de la recherche en dietetique : une publication des Dietetistes du Canada. 2014;75(1):22-8. doi: 10.3148/75.1.2014.22. PubMed PMID: 24606956.
5. Yeasmin SF, Regmi K. A qualitative study on the food habits and related beliefs of pregnant British Bangladeshis. Health care for women international. 2013;34(5):395-415. doi: 10.1080/07399332.2012.740111. PubMed PMID: 23550950.

6. Hussain B, Bardi JN, Fatima T. Pregnancy related cultural food practices among Pakistani women in the UK: a qualitative study. British Journal of Midwifery. 2021;29(7):402-9. doi: 10.12968/bjom.2021.29.7.402. PubMed PMID: 151268444. Language: English. Entry Date: 20210709. Revision Date: 20210712. Publication Type: Article.

7. Chen L, Low Y, Fok D, Han W, Chong Y, Gluckman P, et al. Dietary changes during pregnancy and the postpartum period in Singaporean Chinese, Malay and Indian women: the GUSTO birth cohort study. Public Health Nutrition. 2014;17(9):1930-8. doi: 10.1017/S1368980013001730 https://journals.cambridge.org/action/displayJournal?jid=PHN. PubMed PMID: 20143274189. Chen LingWei (author).

8. Grewal SK, Bhagat R, Balneaves LG. Perinatal beliefs and practices of immigrant Punjabi women living in Canada. Journal of obstetric, gynecologic, and neonatal nursing : JOGNN. 2008;37(3):290-300. doi: 10.1111/j.1552-6909.2008.00234.x. PubMed PMID: 18507600.

9. Groleau D, Soulière M, Kirmayer LJ. Breastfeeding and the cultural configuration of social space among Vietnamese immigrant woman. Health & place. 2006;12(4):516-26. doi: 10.1016/j.healthplace.2005.08.003. PubMed PMID: 16157504.

10. Stewart MM, Whiteford MB. Dietary habits and obstetrical service utilization during pregnancy and lactation among Tai Dam women of central Iowa. Ecology of Food and Nutrition. 1987;20(2):121-42. doi: 10.1080/03670244.1987.9990993. PubMed PMID: 19881405938. Stewart, M. M. (author).

11. Teo C, Chia A, Colega MT, Chen L, Fok D, Pang W, et al. Prospective associations of maternal dietary patterns and postpartum mental health in a multi-ethnic Asian cohort: the Growing up in Singapore towards Healthy Outcomes (GUSTO) Study. Nutrients. 2018;10(3):299. doi: 10.3390/nu10030299 https://www.mdpi.com/2072-6643/10/3/299/htm. PubMed PMID: 20183264536. Teo, C. (author).

12. Essén B, Johnsdotter S, Hovelius B, Gudmundsson S, Sjöberg NO, Friedman J, Ostergren PO. Qualitative study of pregnancy and childbirth experiences in Somalian women resident in Sweden. BJOG : an international journal of obstetrics and gynaecology. 2000;107(12):1507-12. doi: 10.1111/j.1471-0528.2000.tb11676.x. PubMed PMID: 11192108.

13. Qureshi R, Pacquiao DF. Ethnographic Study of Experiences of Pakistani Women Immigrants With Pregnancy, Birthing, and Postpartum Care in the United States and Pakistan. Journal of Transcultural Nursing. 2013;24(4):355-62. doi: 10.1177/1043659613493438. PubMed PMID: 104222011. Language: English. Entry Date: 20130917. Revision Date: 20200708. Publication Type: Journal Article.
