# Supplementary material for: Cultural food practices and sources of nutrition information among pregnant and postpartum migrant women from low- and middle-income countries residing in high income countries: A systematic review
Source: PLoS One. 2024 May 9;19(5):e0303185. doi: 10.1371/journal.pone.0303185 (PMC11081330; doi:10.1371/journal.pone.0303185)
Supplement: S3 Table — (PDF) [file pone.0303185.s004.pdf]

S3A Table. Quality Assessment for Qualitative Studies.

**Author & year**	**Questions**										**Overall Appraisal**
	Congruity between philosophical perspectives and Research Methodology ?	Congruity between research methodology and research questions/objectives?	Congruity between research methodology and data collection methods?	Congruity between research methodology and representation and data analysis?	Congruity between research methodology and result interpretation ?	Statement locating the researcher culturally or theoretically ?	Statement on the Influence of the researcher on the research?	Are participants and their voices adequately represented?	Statement on ethical approval by appropriate body?	Does the conclusion draw flow from the analysis or interpretation of the data?	
Ahlqvist & Wirfält (2000) [1]	Yes	Yes	Yes	Yes	Yes	Unclear	Yes	Yes	Unclear	Yes	Fair
Essén et al. (2000) [2]	Not Applicable	Yes	Yes	Yes	Yes	No	Yes	Yes	Yes	Yes	Fair
Grewal et al. (2008) [3]	Not Applicable	Yes	Yes	Yes	Yes	Yes	Yes	Yes	Yes	Yes	Good
Groleau et al. (2006) [4]	Yes	Yes	Yes	Yes	Yes	Unclear	Yes	Yes	No	Yes	Fair
Higginbottom et al. (2014) [5]	Yes	Yes	Yes	Yes	Yes	Yes	Yes	Yes	Yes	Yes	Good
Higginbottom et al. (2018) [6]	Yes	Yes	Yes	Yes	Yes	Unclear	Unclear	Yes	No	Yes	Fair
Hussain et al. (2021) [7]	Yes	Yes	Yes	Yes	Yes	Yes	Yes	Yes	Yes	Yes	Good
Legault et al. (2014) [8]	Yes	Yes	Yes	Yes	Yes	Yes	Yes	Yes	Yes	Yes	Good
Qureshi et al. (2013)[9]	Yes	Yes	Yes	Yes	Yes	Yes	Yes	Yes	Yes	Yes	Good
Yeasmin et al. (2013) [10]	Yes	Yes	Yes	Yes	Yes	No	Yes	Yes	Yes	Yes	Fair

S3B Table. Quality Assessment for Cross-sectional studies.

**Author & year**	**Questions**								**Overall Appraisal**
	Were the criteria for inclusion clearly defined?	Were the study subjects and the settings described in detail?	Was the exposure measured in a valid and reliable way?	Were the objective, standard criteria used for measurement of the condition?	Were confounding factors identified?	Were strategies to deal with confounding factors stated?	Were the outcomes measured in a valid and reliable way?	Were appropriate statistical analysis used?	
Karki et al. (2022) [11]	Yes	Yes	Yes	Yes	Yes	No	Yes	Unclear	Fair
Lindsay et al. (2014) [12]	Yes	Yes	Yes	Yes	Yes	Unclear	Yes	Yes	Fair
Lindsay et al. (2021) [13]	Yes	Yes	Yes	Yes	Yes	Yes	Yes	Yes	Good
Stewart et al. (1987) [14]	Unclear	Yes	Yes	Yes	No	No	Unclear	No	Poor

S3C Table. Quality Assessment for Cohort Studies.

**Author & year**	**Questions**											**Overall Appraisal**
	Were the two groups similar and recruited from the same population?	Were the exposures measured similarly to assign people to both exposed and unexposed groups?	Was the exposure measured in a valid and reliable way?	Were confounding factors identified?	Were strategies to deal with confounding factors stated?	Were the groups/ participants free of the outcome at the start of the study?	Was the outcomes measured in a valid and reliable way?	Was the follow up time reported and sufficient to be long enough for outcomes to occur?	Was follow-up complete, and if not, were the reasons to loss to follow up described and explored?	Were strategies to address incomplete follow up utilized?	Was appropriate statistical analysis used?	
Chen et al. (2014) [15]	Yes	Not Applicable	Yes	Yes	Yes	Yes	Yes	Yes	Yes	Not Applicable	Yes	Good
Dennis et al. (2024)	Yes	Not Applicable	Yes	Yes	Yes	Yes	Yes	Yes	Yes	Not Applicable	Yes	Good
Teo et al. (2018) [16]	Yes	Not Applicable	Yes	Yes	Yes	Yes	Yes	Yes	Yes	Not Applicable	Yes	Good

Reference

1. Ahlqvist M, Wirfält E. Beliefs concerning dietary practices during pregnancy and lactation. A qualitative study among Iranian women residing in Sweden. Scandinavian journal of caring sciences. 2000;14(2):105-11. PubMed PMID: 12035273.

2. Essén B, Johnsdotter S, Hovelius B, Gudmundsson S, Sjöberg NO, Friedman J, Ostergren PO. Qualitative study of pregnancy and childbirth experiences in Somalian women resident in Sweden. BJOG : an international journal of obstetrics and gynaecology. 2000;107(12):1507-12. doi: 10.1111/j.1471-0528.2000.tb11676.x. PubMed PMID: 11192108.

3. Grewal SK, Bhagat R, Balneaves LG. Perinatal beliefs and practices of immigrant Punjabi women living in Canada. Journal of obstetric, gynecologic, and neonatal nursing : JOGNN. 2008;37(3):290-300. doi: 10.1111/j.1552-6909.2008.00234.x. PubMed PMID: 18507600.

4. Groleau D, Soulière M, Kirmayer LJ. Breastfeeding and the cultural configuration of social space among Vietnamese immigrant woman. Health & place. 2006;12(4):516-26. doi: 10.1016/j.healthplace.2005.08.003. PubMed PMID: 16157504.

5. Higginbottom GMA, Vallianatos H, Forgeron J, Gibbons D, Mamede F, Barolia R. Food choices and practices during pregnancy of immigrant women with high-risk pregnancies in Canada: a pilot study. BMC pregnancy and childbirth. 2014;14:370. doi: 10.1186/s12884-014-0370-6. PubMed PMID: 25467067.

6. Higginbottom GMA, Vallianatos H, Shankar J, Safipour J, Davey C. Immigrant women's food choices in pregnancy: perspectives from women of Chinese origin in Canada. Ethnicity & health. 2018;23(5):521-41. doi: 10.1080/13557858.2017.1281384. PubMed PMID: 28158953.

7. Hussain B, Bardi JN, Fatima T. Pregnancy related cultural food practices among Pakistani women in the UK: a qualitative study. British Journal of Midwifery. 2021;29(7):402-9. doi: 10.12968/bjom.2021.29.7.402. PubMed PMID: 151268444. Language: English. Entry Date: 20210709. Revision Date: 20210712. Publication Type: Article.

8. Legault A, Marquis M. Nutrition information-seeking behaviour of low-income pregnant Maghrebian women. Canadian journal of dietetic practice and research : a publication of Dietitians of Canada = Revue canadienne de la pratique et de la recherche en dietetique : une publication des Dietetistes du Canada. 2014;75(1):22-8. doi: 10.3148/75.1.2014.22. PubMed PMID: 24606956.

9. Qureshi R, Pacquiao DF. Ethnographic Study of Experiences of Pakistani Women Immigrants With Pregnancy, Birthing, and Postpartum Care in the United States and Pakistan. Journal of Transcultural Nursing. 2013;24(4):355-62. doi: 10.1177/1043659613493438. PubMed PMID: 104222011. Language: English. Entry Date: 20130917. Revision Date: 20200708. Publication Type: Journal Article.

10. Yeasmin SF, Regmi K. A qualitative study on the food habits and related beliefs of pregnant British Bangladeshis. Health care for women international. 2013;34(5):395-415. doi: 10.1080/07399332.2012.740111. PubMed PMID: 23550950.

11. Karki R, Perry C, Wilkinson J, Cole G. Prenatal nutrition among Bhutanese refugees in Utah: a pilot study. Journal of Hunger & Environmental Nutrition. 2022;17(4):494-500. doi: 10.1080/19320248.2022.2032898

https://www.tandfonline.com/doi/full/10.1080/19320248.2022.2032898. PubMed PMID: 20220334050. Karki, R. (author).

12. Lindsay KL, Gibney ER, McNulty BA, McAuliffe FM. Pregnant immigrant Nigerian women: an exploration of dietary intakes. Public health. 2014;128(7):647-53. doi: 10.1016/j.puhe.2014.05.001. PubMed PMID: 25065518.

13. Lindsay AC, Le Q, Nogueira DL, Machado MMT, Greaney ML. Sources of information about gestational weight gain, diet and exercise among Brazilian immigrant women living in the USA: a cross-sectional study. Public Health Nutrition. 2021;24(17):5720-9. doi: 10.1017/S1368980021001798 https://www.cambridge.org/core/journals/public-health-nutrition/article/abs/sources-of-information-about-gestational-weight-gain-diet-and-exercise-among-brazilian-immigrant-women-living-in-the-usa-a-crosssectional-study/1B4EA6E81E0EC4B70E922A79A71D313D. PubMed PMID: 20210485027. Lindsay, A. C. (author).

14. Stewart MM, Whiteford MB. Dietary habits and obstetrical service utilization during pregnancy and lactation among Tai Dam women of central Iowa. Ecology of Food and Nutrition. 1987;20(2):121-42. doi: 10.1080/03670244.1987.9990993. PubMed PMID: 19881405938. Stewart, M. M. (author).

15. Chen L, Low Y, Fok D, Han W, Chong Y, Gluckman P, et al. Dietary changes during pregnancy and the postpartum period in Singaporean Chinese, Malay and Indian women: the GUSTO birth cohort study. Public Health Nutrition. 2014;17(9):1930-8. doi: 10.1017/S1368980013001730 https://journals.cambridge.org/action/displayJournal?jid=PHN. PubMed PMID: 20143274189. Chen LingWei (author).

16. Teo C, Chia A, Colega MT, Chen L, Fok D, Pang W, et al. Prospective associations of maternal dietary patterns and postpartum mental health in a multi-ethnic Asian cohort: the Growing up in Singapore towards Healthy Outcomes (GUSTO) Study. Nutrients. 2018;10(3):299. doi: 10.3390/nu10030299 https://www.mdpi.com/2072-6643/10/3/299/htm. PubMed PMID: 20183264536. Teo, C. (author).
